# Density-based fractionation of soil organic matter: effects of heavy liquid and heavy fraction washing

**DOI:** 10.1038/s41598-019-46577-y

**Published:** 2019-07-12

**Authors:** César Plaza, Beatrice Giannetta, Iria Benavente, Costantino Vischetti, Claudio Zaccone

**Affiliations:** 1 Instituto de Ciencias Agrarias, Consejo Superior de Investigaciones Científicas, Serrano 115 bis, 28006 Madrid, Spain; 2 Department of Agricultural, Food and Environmental Sciences, Polytechnic University of Marche, via Brecce Bianche 10, 60131 Ancona, Italy; 3 Departamento de Producción Agraria, ETSI Agronómica, Alimentaria y de Biosistemas, Universidad Politécnica de Madrid, Ciudad Universitaria, 28040 Madrid, Spain; 4 Department of the Sciences of Agriculture, Food and Environment, University of Foggia, via Napoli 25, 71122 Foggia, Italy

**Keywords:** Carbon cycle, Carbon cycle, Environmental chemistry, Environmental chemistry

## Abstract

Physical fractionation methods used in soil organic matter (SOM) research commonly include density-based procedures with heavy liquids to separate SOM pools with varying turnover rates and functions. Once separated, the heavy SOM pools are often thoroughly rinsed with water to wash off any residues of the heavy liquids. Using four soils with contrasting properties, we investigated the effects of using either sodium polytungstate (SPT) or sodium iodide (NaI), two of the most commonly used heavy liquids, on the distribution of organic carbon (C) and total nitrogen (N) in free light, intra-aggregate light, and mineral-associated heavy SOM pools isolated by a common fractionation scheme. We also determined the effects of washing the mineral-associated heavy SOM fractions on the recovery of organic C and total N after separation. Because of its smaller viscosity compared to that of NaI, SPT consistently yielded greater intra-aggregate and smaller mineral-associated soil organic C contents. We also confirm that some commercial SPT products, such as the one used here, can contaminate organo-mineral heavy pools with N during density-based fractionation procedures. We do not recommend the repeated washing of heavy fractions separated with Na-based heavy liquids, as this can mobilize SOM.

## Introduction

Soil organic matter (SOM) is key to terrestrial ecosystems, contributing to the support of natural vegetation and agricultural production, filtering and holding water, and storing carbon (C)^1,2^. The preservation of SOM is of paramount importance because of the need to maintain these ecosystem functions and services to face major global issues, such as food security, desertification, and climate change^3,4^. Key mechanisms controlling the preservation of SOM include occlusion within soil aggregates and sorption onto mineral surfaces, which limit the accessibility of SOM to decomposers and enzymes^3,5–7^.

The acknowledge of the importance of these preservation mechanisms has led to the increased use of physical fractionation methods to isolate SOM pools of distinct location within the soil mineral matrix^5,8,9^. Among the large number of physical fractionation methods available in the literature (see Poeplau *et al*.^9^ for a recent comparative description of many of them), the density-based separation scheme developed by Golchin *et al*.^10^ is one of the most commonly used and has constituted the basis for the development of other fractionation methods^6,11–17^. This scheme is intended to separate three fractions: a free light SOM fraction not physically disconnected from microorganisms and enzymes; a occluded light SOM fraction located within aggregates, which forms a physical barrier that limits O_2_ diffusion and the accessibility of microorganisms and enzymes; and a heavy fraction consisting of SOM intimately associated with minerals, which decreases microbial and enzymatic capacity to decompose organic substrates^10^. Briefly, the free SOM fraction is isolated by an initial density separation, and the intra-aggregate SOM is then separated from the mineral-associated pool by a second density separation after ultrasonic disruption of stable aggregates^10^.

Sodium polytungstate (SPT, Na_6_[H_2_W_12_O_40_]) and sodium iodide (NaI) are the chemical reagents most commonly used to prepare the heavy liquids for density-based fractionation of SOM^6,9–17^. After separating the light SOM fractions, the mineral-associated heavy SOM fractions are commonly washed with deionized water to eliminate the remaining SPT and NaI. Having different properties, these heavy liquids may interact differently with the soil and therefore yield different fractionation results. Similarly, the washing intended to eliminate SPT and NaI may arguably cause solubilization and loss of mineral-associated SOM because of hydrolysis reactions and increased pH^18,19^, thus affecting the recovery of SOM after fractionation. In spite of the potential importance of these effects, there is a lack of studies in the literature specifically and systematically addressing these issues, which may seriously compromise meaningful interpretations of SOM dynamics.

Here we (a) investigated comparatively the effects of two heavy liquids, SPT versus NaI, on the distribution of organic C and total N in free, intra-aggregate, and mineral-associated pools separated by a common density-based procedure similar to the one described by Golchin *et al*.^10^; (b) determined the effects of washing the mineral-associated heavy SOM fraction isolated using either SPT or NaI on its organic C and total N contents; and (c) evaluated the influence of soil properties on the potential effects of the heavy liquid and washing. To do this, we used four samples collected from the surface layer of four contrasting soils (SOIL 1 to 4), with the aim of covering a wide range of textures (Table 1)^17,20,21^. The final purpose of this study was to provide general recommendations to prevent misinterpretation of fractionation results in SOM research.

**Table 1:** Location, vegetation, class, sampling depth, and main physical and chemical properties of the soils used in this study (data from Giannetta *et al*.^17^ and Jiménez-González *et al*.^20,21^).

Soil	Location	Vegetation	Class	Depth (cm)	Texture	Sand (g kg^−1^)	Silt (g kg^−1^)	Clay (g kg^−1^)	pH	EC ^a^ (dS m^−1^)	CEC ^b^ (cmol_c_ kg^−1^)	Organic C (g kg^−1^)	Total N (g kg^−1^)
SOIL 1	El Berrueco, Madrid, Spain	*Paeonia coriacea*	Dystric Cambisol	0–10	Sandy loam	522	352	126	5.3	0.418	14.4	45	3.7
SOIL 2	Foresta Cesane, Fossombrone, Italy	*Pinus nigra*	Rendzic Leptosol	0–10	Loam	379	481	140	8.3	0.064	22.1	31	2.2
SOIL 3	Chiaserna, Cantiano, Italy	*Pinus nigra*	Haplic Cambisol	0–20	Clay loam	380	232	388	8.0	0.059	49.6	57	4.6
SOIL 4	Castelluccio, Castelsantangelo sul Nera, Italy	*Fagus sylvatica*	Haplic Cambisol	0–20	Clay	265	312	423	8.1	0.082	35.9	35	2.4

^a^Electrical conductivity. ^b^Cation exchange capacity (determined according to Sparks *et al*.^29^).

## Results

The SPT used here to prepare the heavy liquid for density fractionations contained no detectable amounts of C but 0.646 g kg^−1^ of total N. The NaI powder had no detectable amounts of total C and N. The SPT solution had a viscosity of 1.1 mPa s, a pH of 3.3, and an electrical conductivity of 61 dS m^−1^ whereas the NaI liquid had a viscosity of 1.7 mPa s, a pH of 8.0, and an electrical conductivity of 154 dS m^−1^.

**Figure 1:**
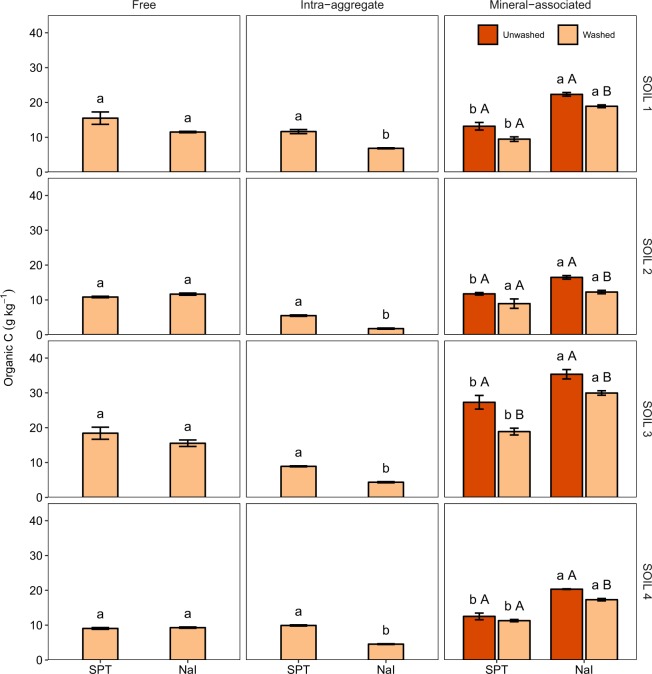
Free, intra-aggregate, and mineral-associated organic C content (mean ± standard error) of soils (SOIL 1 to 4) as affected by the heavy liquid used for separation (sodium polytungstate, SPT, vs. sodium iodide, NaI) and washing. Within the same soil, fraction, and washing treatment, different lowercase letters indicate statistically significant differences at the 0.05 level. Within the same soil and heavy liquid, different uppercase letters indicate statistically significant differences at the 0.05 level.

**Figure 2:**
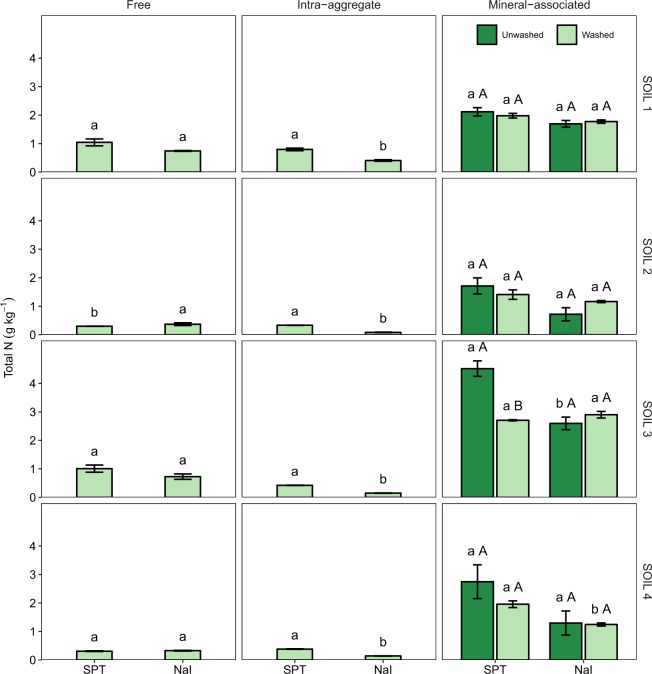
Free, intra-aggregate, and mineral-associated N content (mean ± standard error) of soils (SOIL 1 to 4) as affected by the heavy liquid used for separation (sodium polytungstate, SPT, vs. sodium iodide, NaI) and washing. Within the same soil, fraction, and washing treatment, different lowercase letters indicate statistically significant differences at the 0.05 level. Within the same soil and heavy liquid, different uppercase letters indicate statistically significant differences at the 0.05 level.

Analysis of variance tests revealed significant main and interaction effects (*P* < 0.001) of the heavy liquid and soil on free organic C and total N contents (Table S1). Post hoc pairwise tests, however, being more conservative, failed to detect significant differences between the effects of SPT and NaI on free organic C and total N contents for any of the soils examined (Figs 1 and 2). Nonetheless, compared to SPT, NaI tended to yield slightly smaller free organic C and total N contents for the soils with the largest total organic C and total N contents, SOIL 1 and SOIL 3 (Table 1).

Intra-aggregate organic C and N contents and C/N ratio were significantly affected (*P* < 0.001) by the heavy liquid and soil factors and their interaction (Tables S1). Compared to SPT, NaI resulted in significantly smaller intra-aggregate organic C and total N contents for the four soils examined, with the magnitude of the differences slightly depending on the soil (Figs 1 and 2).

We also found significant two-way interaction effects of the heavy liquid and soil (*P* < 0.001) and the washing and soil (*P* = 0.004) on mineral-associated organic C content, and significant three-way interaction effects of the heavy liquid, washing, and soil on mineral-associated N content (*P* = 0.007) (Table S1). Specifically, with respect to SPT, NaI resulted in significantly larger mineral-associated organic C content for the four soils, especially for SOIL 1 and SOIL 3, regardless of the washing treatment (Fig. 1). Independently of the heavy liquid used, washing decreased mineral-associated organic C content, especially for SOIL 3. Compared to NaI, SPT yielded larger mineral-associated N content (Fig. 2). The size and significance of these differences depended on the soil. For SPT treated soils, washing decreased mineral-associated N content, but had a negligible effect for NaI treated soils (Fig. 2).

Total organic C recovery after fractionation was significantly affected by the interaction of the washing and soil (*P* = 0.026) (Table S1). In particular, washing decreased organic C recovery by 0.2% for SOIL 1 to 21% for SOIL 3 (Table 2). We also found significant interaction effects of the washing and soil (*P* = 0.022) and the heavy liquid and washing (*P* < 0.001) on total N recovery (Table S1). Specifically, N recovery after SOM separation with SPT and without washing the mineral-associated fraction ranged from 106 (SOIL 1) to 143% (SOIL 4) (Table 2). With washing, N recovery after SOM separation with SPT decreased (especially for SOIL 3 and SOIL 4), ranging from 88 to 110%. Total N recovery with NaI was markedly smaller and not significantly affected by the washing treatment (Table 2).

**Table 2:** Organic C and total N recovery (mean ± standard error) for each soil (SOIL 1 to 4) after fractionation, as affected by the heavy liquid used for separation (sodium polytungstate, SPT, vs. sodium iodide, NaI) and washing.

Recovery	Heavy liquid	Washing	SOIL 1	SOIL 2	SOIL 3	SOIL 4
Organic C (%)	SPT	Unwashed	85.2 ± 6.3 a A	92.5 ± 0.9 a A	98.1 ± 0.1 a A	87.8 ± 3.3 a A
	SPT	Washed	85.0 ± 7.3 a A	82.3 ± 3.2 a A	77.3 ± 3.2 a B	86.3 ± 2.3 a A
	NaI	Unwashed	89.9 ± 1.0 a A	98.3 ± 3.4 a A	97.5 ± 1.9 a A	96.4 ± 1.2 a A
	NaI	Washed	82.5 ± 1.2 a B	83.8 ± 3.2 a B	85.3 ± 2.1 a B	87.7 ± 1.0 a A
Total N (%)	SPT	Unwashed	105.5 ± 9.1 a A	107.1 ± 12.8 a A	131.4 ± 1.1 a A	143.1 ± 25.2 a A
	SPT	Washed	104.7 ± 6.2 a A	92.2 ± 7.9 a A	87.8 ± 2.5 a B	110.4 ± 3.8 a A
	NaI	Unwashed	76.2 ± 1.8 a A	51.5 ± 10.7 b A	77.1 ± 3.8 b A	73.5 ± 18.5 a A
	NaI	Washed	79.7 ± 1.6 b A	75.4 ± 4.8 a A	80.5 ± 5.5 a A	71.5 ± 2.6 b A

Within the same soil and washing treatment, different lowercase letters indicate statistically significant differences at the 0.05 level. Within the same soil and heavy liquid, different uppercase letters indicate statistically significant differences at the 0.05 level.

## Discussion

Our research highlights substantial differences between the heavy liquids in SOM fractionation results. These differences may be mainly related to the significantly different physicochemical and chemical properties of the heavy liquids, while only secondarily depend on the specific properties of the soil under study. Despite the observed interaction effects of the heavy liquid and soil, our results do not point to clear relationships between such interactions and soil texture or any other specific soil properties. The effects of the heavy liquid tend to dominate and be consistent across soils.

The no significant differences found between the effects of SPT and NaI on free organic C and total N contents for any of the soils examined is consistent with previous observations^13^. The greater intra-aggregate and smaller mineral-associated organic C contents obtained after fractionation with SPT, compared to NaI, which is in general agreement with previous works^14,15^, may be attributed to the smaller viscosity of the SPT solution. Smaller viscosities favor the propagation of the ultrasonic pulses and the cavitation process^22^. This may increase the effectiveness of the ultrasonic disruption treatment to break up aggregates and the release of intra-aggregate SOM, which in turn decreases the amount of SOM recovered in the mineral-associated pool.

Because of the lower viscosity and higher efficacy of sonication, SPT also results in greater intra-aggregate N compared to NaI, but not in smaller mineral-associated N. This is because, unlike NaI, the SPT powder used to prepared the heavy solution is contaminated with N as supplied. Some of the N in SPT inevitably ends up in the heavy fraction remaining after the last density separation, thus adding up to the mineral-associated N pool. The concentration of N in the SPT used here is within the range detected in commercially available standard SPT for the preparation of heavy liquid in a previous study^23^. Conversely, low N content SPT, which can be purchased from some manufacturers, has been shown not to adversely contaminate soils after density fractionation^23^.

**Figure 3:**
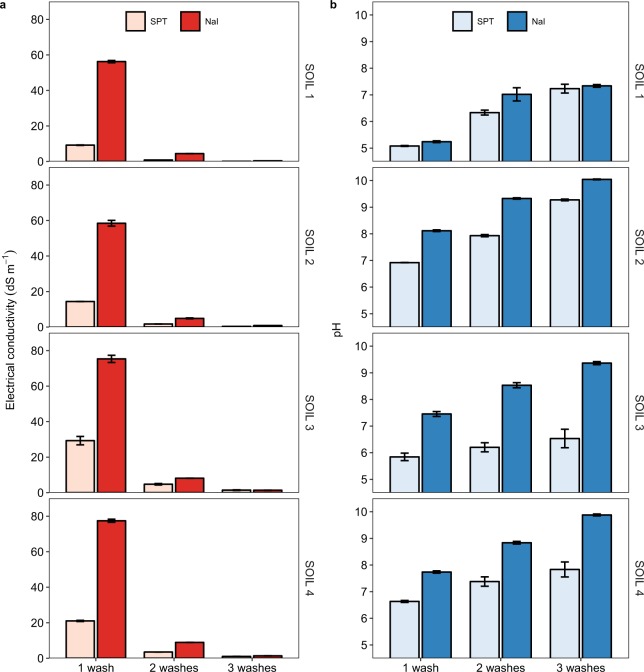
Electrical conductivity and pH of wash water after 1, 2, and 3 washes of the mineral-associated organic matter fraction of the soils used in this study (SOIL 1 to 4), as affected by the heavy liquid used for separation (sodium polytungstate, SPT, vs. sodium iodide, NaI).

To remove SPT and NaI residues, the organo-mineral heavy fractions remaining after density-based separations are often thoroughly rinsed with water. Our results show that this procedure may result in significant losses of organic C from the mineral-associated SOM fraction, thus hampering the interpretation of fractionation results. These C losses, which tend to become more evident with increasing the number of washes and soil organic C content (Supplementary Fig. S1), can be attributed to soil dispersion and organic matter solubilization induced by the high amounts of Na added with the SPT and NaI solutions. Initially, the high electrolyte concentration in the heavy solutions helps maintain soil aggregated during density fractionation^19^. With washing, this coagulation effect of a high electrolyte concentration disappears (Fig. 3a), and soil particles disperse. Washing Na-saturated soils causes hydrolysis reactions and increased pH (Fig. 3b). This rise in pH may also cause the disruption of bonds between organic matter and minerals and the conversion of acidic organic components to their soluble salt forms^18,19^. This may help explain why total organic C recovery from SOIL 1, which has the lowest pH, decreases with washing to a lesser extent than does the total organic C recovery from the other soils examined.

As a whole, our results reveal substantial effects of the heavy liquid used for density fractionation on the distribution of organic C and total N in intra-aggregate and mineral associated pools. These effects are largely independent of the soil and have several implications that need to be accounted for when applying SOM fractionation methods and interpreting SOM fractionation data. First, to facilitate the comparison of results across studies, ultrasonic energy has to be properly calibrated not only for the soil under study but also for the heavy liquid and applied to an extent to completely disrupt aggregates. Our results also highlight the need of determining C and N levels in the heavy liquids used for density-based SOM separations, particularly N in SPT, to prevent or account for potential contamination effects. Finally, we do not recommend repeated washing of the organo-mineral heavy soil fractions separated with Na-based heavy liquids, as this can lead to mobilization of SOM.

Consistently with methods commonly used in previous studies, here we used heavy liquids at a density of 1.85 g mL^−1^ ^11,24–26^ and soil samples passed through a 2-mm sieve^9,10^. Future research should test other densities, and consider the use of soil samples sieved through larger mesh sizes, to further improve our understanding about the impact of heavy liquids and washing on the distribution of organic C and total N in SOM fractions. Here we only focused on the effects of washing the mineral associated heavy SOM fraction. The effects of washing the free and intra-aggregate light SOM fractions also need to be investigated in future studies.

## Methods

### Soil samples

We collected four samples from the surface layer of four contrasting soils (SOIL 1 to 4), with the aim of covering a wide range of textures^17,20,21^. The samples were air dried, gently crushed, and sieved to 2 mm.

### Soil organic matter fractionation

The prepared soil samples (dried and 2-mm sieved) were subjected to the physical fractionation scheme developed by Golchin *et al*.^10^ to isolate free, intra-aggregate, and mineral-associated SOM. The fractionation was conducted using a solution of either SPT or NaI as heavy liquid and either not washing or washing the isolated mineral-associated SOM fraction. Specifically, 80 mL of either SPT (purum p.a., for the preparation of heavy liquid, for sink-float analysis, ≥85% WO_3_ basis, Fluka, Sigma-Aldrich, St. Luois, MO) or NaI (ACS reagent, ≥99.5%, Sigma-Aldrich, St. Luois, MO) at a density of 1.85 g mL^−1^ was added to 10 g of soil in a 100-mL centrifuge tube. The centrifuge tube was rotated at 1 revolution s^−1^ for 30 s in an overhead shaker to allow free SOM outside aggregates to float. After centrifugation at 2500 g for 30 min, the floating light fraction (free SOM) was separated from the heavy fraction by suction and filtration through a glass fiber filter (GF/A, Whatman, UK) and washed thoroughly with deionized water. The heavy fraction in the centrifuge tube was resuspended and dispersed in the SPT or NaI solution by sonication at an energy input of 1500 J g^−1^. The floating light fraction (intra-aggregate SOM) was separated from the heavy fraction (mineral-associated SOM) by centrifugation at 2500 g for 60 min, suction, and filtration through a glass fiber filter, and washed thoroughly with deionized water. The fractionation procedure was repeated six times for each soil and heavy liquid. Three of the six replicates of the isolated mineral-associated SOM were not washed, and the other three replicates were thoroughly washed three times by adding 80 mL of deionized water, shaking for 10 min, and centrifuging at 2500 g for 30 min.

### Chemical and physicochemical analysis

The soils and SOM fractions were analyzed for organic C and total N content by dry combustion using a Thermo Flash 2000 NC Soil Analyzer. Prior to organic C analysis, the soils and mineral-associated SOM fractions were subjected to acid fumigation to remove carbonates^27^.

The SPT and NaI reagents (powders) used to prepare the solutions for density fractionations were analyzed for total C and N by dry combustion, as described above for the soils and SOM fractions. The prepared heavy liquids were analyzed for viscosity, pH, and electrical conductivity at 20 °C using a capillary viscometer, a pH meter, and a conductivity meter, respectively.

### Data analysis

We used two-way analysis of variance (ANOVA) tests to evaluate the main and interaction effects of the heavy liquid and soil on free and intra-aggregate organic C and total N, and three-way ANOVA tests to evaluate the effects of the heavy liquid, washing, and soil on mineral-associated organic C and total N and on the recovery of each element after fractionation. When assumptions of normality and homoscedasticity were not met, we used nonparametric ANOVA on aligned rank transformed data. Post hoc pairwise comparisons within each soil were conducted using t or Wilcoxon rank-sum tests at the 0.05 level. All data analyses were performed using R statistical software version 3.5.1^28^.

## Supplementary information

Supplementary Information

## Data Availability

The data and R scripts related to this study are available from the corresponding author upon request.
