# Supplementary material for: Density-based fractionation of soil organic matter: effects of heavy liquid and heavy fraction washing
Source: Sci Rep. 2019 Jul 12;9:10146. doi: 10.1038/s41598-019-46577-y (PMC6626057; doi:10.1038/s41598-019-46577-y)
Supplement: Supplementary file 1 — Supplementary Information [file 41598_2019_46577_MOESM1_ESM.pdf]

Supplementary Information

Density-based fractionation of soil organic matter: effects of heavy liquid and heavy fraction washing

César Plaza, Beatrice Giannetta, Iria Benavente, Costantino Vischetti, Claudio Zaccone

This file includes Table S1 and Figure S1

Table S1

Analysis of variance for free, intra-aggregate, and mineral-associated organic C and total N content and total recovery, as affected by heavy liquid (HL), washing (WA), and soil type (SO).

	Free organic C	Intra-aggregate organic C	Mineral-associated organic C	Free N	Intra-aggregate N	Mineral-associated N	Recovery of organic C	Recovery of N
HL	<0.001***	<0.001***	<0.001***	<0.001***	<0.001***	<0.001***	0.043*	<0.001***
WA	-	-	<0.001***	-	-	0.020*	<0.001***	0.026*
SO	<0.001***	<0.001***	<0.001***	<0.001***	<0.001***	<0.001***	0.261	0.061
HL × WA	-	-	0.974	-	-	<0.001***	0.436	<0.001***
HL × SO	<0.001***	<0.001***	<0.001***	<0.001***	<0.001***	0.046*	0.852	0.121
WA × SO	-	-	0.004**	-	-	0.012*	0.026*	0.022*
HL × WA × SO	-	-	0.223	-	-	0.007**	0.264	0.091

* $P < 0.05$; ** $P < 0.01$; *** $P < 0.001$.

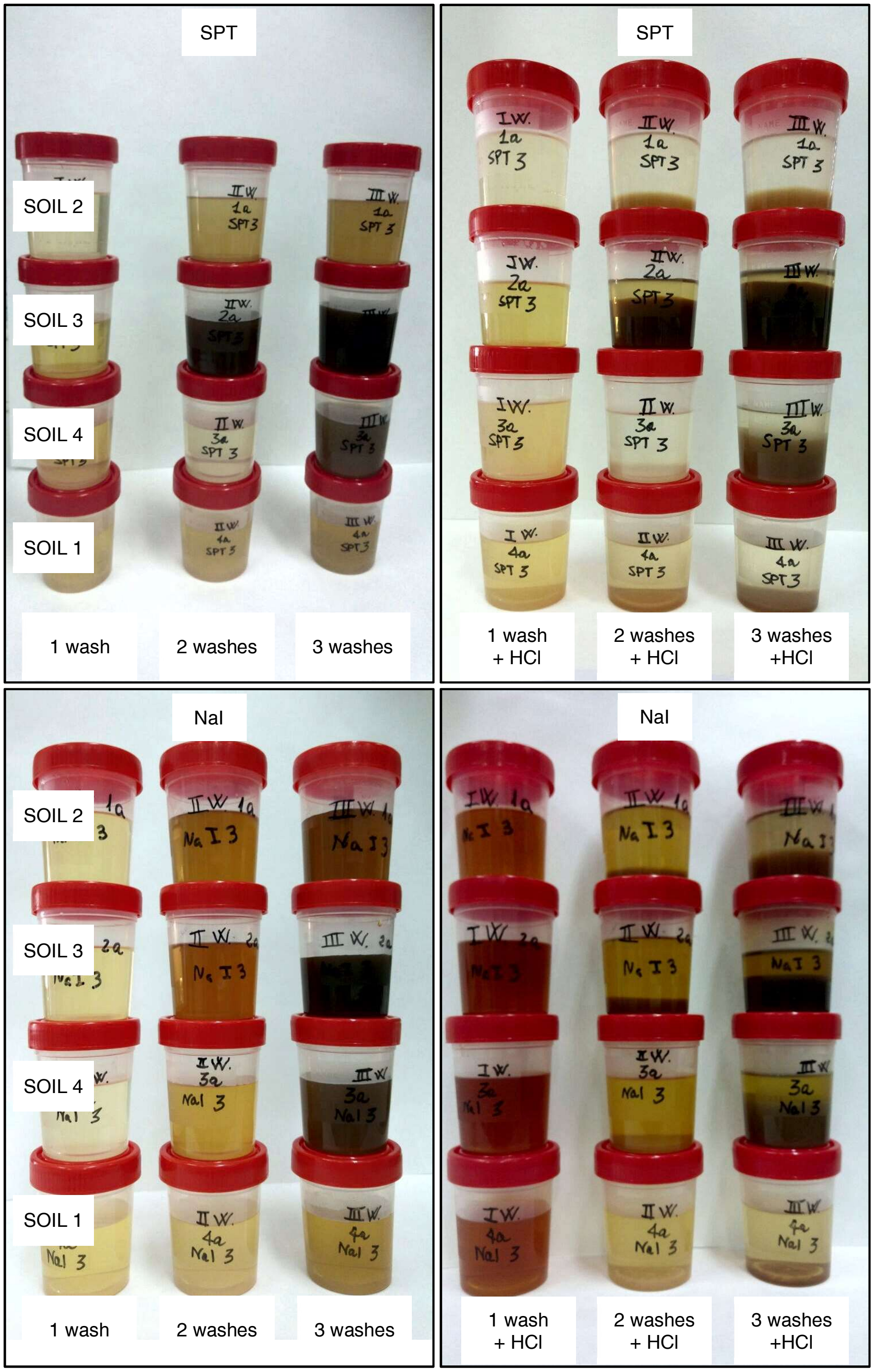

Figure S1. Wash water after 1, 2, and 3 washes of the mineral-associated organic matter fraction separated with either sodium polytungstate (SPT) or sodium iodide (NaI) of the soils used in this study (SOIL 1 to 4) before and after acidification with HCl (pictures taken by Beatrice Giannetta).
